# Response of atmospheric pCO$_2$ to a strong AMOC weakening under low and high emission scenarios

**DOI:** 10.1007/s00382-024-07295-y

**Published:** 2024-06-06

**Authors:** Amber A. Boot, Anna S. von der Heydt, Henk A. Dijkstra

**Affiliations:** 1 Department of Physics, Institute for Marine and Atmospheric Research Utrecht, Utrecht University, Princetonplein 5, Utrecht, 3584CC The Netherlands; 2 Centre for Complex Systems Studies, Utrecht University, Leuvenlaan 4, Utrecht, 3584CE The Netherlands

**Keywords:** Atlantic meridional overturning circulation, Carbon cycle, AMOC weakening, Climate change, Marine biosphere, Atmospheric pCO$_2$

## Abstract

The Earth System is warming due to anthropogenic greenhouse gas emissions which increases the risk of passing a tipping point in the Earth System, such as a collapse of the Atlantic Meridional Overturning Circulation (AMOC). An AMOC weakening can have large climate impacts which influences the marine and terrestrial carbon cycle and hence atmospheric pCO$_2$. However, the sign and mechanism of this response are subject to uncertainty. Here, we use a state-of-the-art Earth System Model, the Community Earth System Model v2 (CESM2), to study the atmospheric pCO$_2$ response to an AMOC weakening under low (SSP1-2.6) and high (SSP5-8.5) emission scenarios over the years 2015–2100. A freshwater flux anomaly in the North Atlantic strongly weakens the AMOC, and we simulate a weak positive pCO$_2$ response of 0.45 and 1.3 ppm increase per AMOC decrease in Sv for SSP1-2.6 and SSP5-8.5, respectively. For SSP1-2.6 this response is driven by both the oceanic and terrestrial carbon cycles, whereas in SSP5-8.5 it is solely the ocean that drives the response. However, the spatial patterns of both the climate and carbon cycle response are similar in both emission scenarios over the course of the simulation period (2015–2100), showing that the response pattern is not dependent on cumulative CO$_2$ emissions up to 2100. Though the global atmospheric pCO$_2$ response might be small, locally large changes in both the carbon cycle and the climate system occur due to the AMOC weakening, which can have large detrimental effects on ecosystems and society.

**Supplementary Information:**

The online version contains supplementary material available at 10.1007/s00382-024-07295-y.

## Introduction

Anthropogenic emissions of greenhouse gases cause the Earth System to change and warm up. As temperatures increase, we are at risk of crossing tipping points with possibly large detrimental effects on our climate, biodiversity and human communities (Lenton et al. 2008; McKay et al. 2022). One of these tipping points can occur in the Atlantic Meridional Overturning Circulation (AMOC) (Lenton et al. 2008). Currently, the AMOC is in a so-called on-state where it transports heat from the Southern Hemisphere to the Northern Hemisphere and thereby modulates global and especially European climate (Buckley and Marshall 2016). In models, the AMOC can be strongly weakened and in this so-called collapsed state (or off-state), the northward heat transport is disrupted with large global climatic effects (Orihuela-Pinto et al. 2022).

Proxy-based evidence suggest that AMOC collapses have occurred frequently during the Pleistocene where they are a main source of millennial variability (e.g. the Dansgaard-Oeschger cycles; Rahmstorf 2002; Lynch-Stieglitz 2017). The disrupted heat transport causes warming of surface air temperature (SAT) and sea surface temperature (SST) in the Southern Hemisphere, while the Northern Hemisphere cools (also called the ‘bipolar seesaw’; Vellinga and Wood 2002; Caesar et al. 2018), with local SAT changes up to 10$^{\circ }$C (Cuffey and Clow 1997; Rahmstorf 2002). In models, the bipolar seesaw results in an increased northern hemispheric sea-ice extent and changes in atmospheric dynamics (Vellinga and Wood 2002; Orihuela-Pinto et al. 2022). The changes in atmospheric dynamics are, for example, seen in wind fields with strengthened trade winds and strengthened Pacific Walker Circulation (Orihuela-Pinto et al. 2022), and a southward shift of the Intertropical Convergence Zone (ITCZ) (Zhang and Delworth 2005; Jackson et al. 2015). The tipping threshold for the AMOC is estimated to be around 4 $^{\circ }$C of warming relative to pre-industrial climate (McKay et al. 2022).

In addition to the climate system, also the carbon cycle is affected by an AMOC collapse. In the ocean, the change in ocean circulation affects the advection of important tracers such as Dissolved Inorganic Carbon (DIC) and nutrients (Zickfeld et al. 2008). An AMOC collapse can also change upwelling rates and surface stratification, processes that are important for driving Net Primary Production (NPP) and carbon sequestration in the deep ocean. Terrestrial primary productivity is affected by the changing temperature and precipitation patterns. Locally, this can lead to both a reduction or an increased uptake of CO$_2$ (e.g. Köhler et al. 2005). Several studies have looked into a potential feedback between AMOC dynamics and atmospheric pCO$_2$, which is controlled by the exchange of the atmosphere with the ocean and land carbon stocks. These studies (e.g. Marchal et al. 1998; Köhler et al. 2005; Schmittner and Galbraith 2008), mostly focused on Pleistocene and pre-industrial conditions, show a wide range of possible responses. There is no clear consensus on the responses of the terrestrial and ocean carbon stock to an AMOC weakening, or to the net effect on atmospheric pCO$_2$, which can be attributed to different climatic boundary conditions, timescales assessed, and model detail used (Gottschalk et al. 2019). In CMIP6 models, the AMOC gradually weakens up to 2100 and, independent of the used emission scenario (Weijer et al. 2020), no AMOC tipping is found. However, these models are thought to be biased towards a too stable AMOC (e.g. Cheng et al. 2018; Weijer et al. 2019), and a recent observation based study has indicated that the AMOC may tip between 2025 and 2095 (Ditlevsen and Ditlevsen 2023).

The carbon cycle is also affected by climate change. In the ocean, the effect on the solubility pump is relatively straight forward: increased warming, and increased CO$_2$ concentrations, reduce ocean pH and the solubility of CO$_2$, which reduces the uptake capacity of the ocean (Sarmiento et al. 1998). The biological pump in Coupled Model Intercomparison Project 6 (CMIP6; Eyring et al. 2016) models is much more uncertain though (Henson et al. 2022; Wilson et al. 2022), especially given that the spread in NPP and Export Production (EP) has increased from CMIP5 to CMIP6 (Kwiatkowski et al. 2020; Tagliabue et al. 2021). The terrestrial biosphere is affected for example through increased primary production related to CO$_2$ fertilization (Zhu et al. 2022), but also increased respiration due to permafrost melt (Burke et al. 2020).

Studies looking at the combined effect of strong AMOC weakening and anthropogenic climate change on the future carbon cycle are limited. A projected AMOC weakening affects both the solubility and the biological carbon pumps (Liu et al. 2023), and generally leads to reduced uptake of (anthropogenic) carbon in the ocean (Obata 2007; Zickfeld et al. 2008; Liu et al. 2023), which can be partially compensated for by the terrestrial biosphere (Zickfeld et al. 2008). However, the net effect has been found to be small due to competing effects (Swingedouw et al. 2007; Zickfeld et al. 2008). Though global effects might be weak, local effects can be quite strong. For example, a weakening of the AMOC can also result in a local reduction in primary productivity (Whitt and Jansen 2020), changes in the plankton stock (Schmittner 2005) and plankton composition (Boot et al. 2023), which all can lead to reduced CO$_2$ uptake of the ocean (e.g. Yamamoto et al. 2018; Boot et al. 2023). These local changes related to an AMOC weakening are strongest in the Atlantic Ocean (Katavouta and Williams 2021).

The novel aspect of this paper is that we consider the effect of AMOC weakening on the carbon cycle under climate change in a state-of-the-art global climate model, the Community Earth System Model v2 (CESM2; Danabasoglu et al. 2020), as explained in Sect. 2. We use a strong freshwater forcing in the North Atlantic to artificially weaken the AMOC and consider two different emission scenarios, Shared Socioeconomic Pathways (SSPs), with low (SSP1-2.6) and high (SSP5-8.5) emissions (O’Neill et al. 2020). In the results of Sect. 3 and the subsequent analysis, we focus on the mechanisms how a forced AMOC weakening affects atmospheric pCO$_2$ under climate change.

## Method

In the CESM2 (Danabasoglu et al. 2020), the atmosphere is represented by the CAM6 model, the land by the CLM5 model (Lawrence et al. 2019), sea ice by the CICE model, ocean circulation by POP2 (Smith et al. 2010), and ocean biogeochemistry by MARBL (Long et al. 2021). The ocean models POP2 and MARBL are both run on a displaced Greenland pole grid at a nominal horizontal resolution of 1$^{\circ }$, with 60 non-equidistant vertical levels. The ocean biogeochemical module MARBL is based on a NPZD-model, where four nutrients (N, P, Fe, and Si) together with light co-limit the production of three phytoplankton groups (diatoms, diazotrophs and small phytoplankton) which are grazed upon by one zooplankton group. The terrestrial carbon cycle is represented with CLM5. This module represents several surface processes such as biogeochemistry, ecology, human influences, biogeophysics and the hydrological cycle. As we use the default CESM2 version, there is no dynamic vegetation. For a complete overview of the CESM2 model and submodules we refer the reader to Danabasoglu et al. (2020) (CESM2), Long et al. (2021) (MARBL), and Lawrence et al. (2019) (CLM5).

We performed emission forced CESM2 simulations with two different emission scenarios, the low emission scenario SSP1-2.6 (126) and the high emission scenario SSP5-8.5 (585). For each emission scenario, a control (CTL) and a hosing (HOS) simulation were carried out. The CTL simulations were only forced with the greenhouse gas emissions, while the HOS simulations were forced with greenhouse gas emissions and an additional, artificial freshwater flux in the North Atlantic. This freshwater forcing is uniformly distributed in the North Atlantic Ocean over the latitudes 50–70$^\circ$ N (Fig. S1), and is kept constant at a rate of 0.5 Sv, a typical value for these type of experiments (see e.g. Orihuela-Pinto et al. 2022; Jackson et al. 2023), over the entire simulation period. Both the alkalinity and DIC of the freshwater forcing are set to zero, meaning that the freshwater forcing dilutes the alkalinity and DIC concentrations of the surface.

We will refer to the simulations by their simulation type (CTL or HOS) and the respective emission scenario (126 or 585), e.g. as CTL-126 and HOS-585. All simulations are run from year 2015 to year 2100 and are initialized by values of the NCAR CMIP6 emission driven historical simulation (Danabasoglu 2019). The used model output is based on monthly means, and line plots are smoothed with a 5 year running mean. When looking at the difference between the HOS and CTL simulations, we subtract the CTL simulations from the HOS simulations.

In Sect. 3, we look at relations between variables such as the gas exchange and NPP. To test the relation between such variables, we perform a linear regression on the decadal trends of these variables. We do this analysis on the difference between the HOS and CTL simulations, i.e. we do not perform this analysis for the individual simulations. We determine decadal trends for each decade starting from the 2020’s to the 2090’s, resulting in 8 data points. The decadal trend of the 2020’s is determined as the difference between the average over 2021 to 2030 and the average over 2016 to 2020. The decadal trend of the 2030’s is determined as the difference between the average over 2031 to 2040 and the average over 2021 and 2030. The other decadal trends are determined in a similar fashion. This analysis provides us with the sign and strength of the relation between two variables, and the statistical significance of the relation. The regions for which a linear regressions are determined can be seen in Fig. S2, and a result of this analysis for the subtropical North Atlantic Ocean can be seen in Fig. S3.

## Results

### Climate reponse

**Fig. 1:**
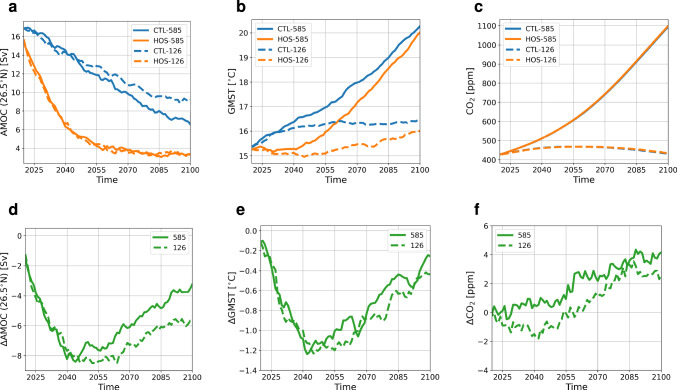
**a** AMOC strength at 26.5$^{\circ }$N in Sv, **b** GMST in $^{\circ }$C, and **c** atmospheric CO$_2$ concentration in ppm for the period 2020–2100 smoothed with a 5 year moving mean. In (**a**–**c**) blue lines represent the control (CTL) simulations, and orange lines the HOS simulations. (**d**–**f**) as in (**a**–**c**) but for the difference between the HOS simulations and the control simulations. In all subplots dashed lines represent SSP1-2.6 (126) and solid lines SSP5-8.5 (585)

**Fig. 2:**
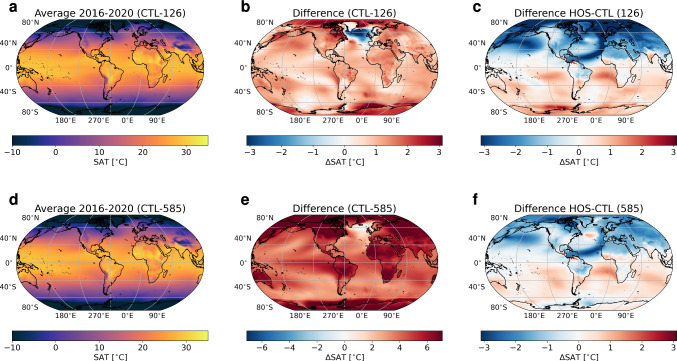
Results for Surface Air Temperature (SAT) in $^{\circ }$C. The top row (**a**–**c**) is for SSP1-2.6, and the bottom row (**d**–**f**) for SSP5-8.5. The left column (**a**, **d**) represents the average over 2016–2020 in the control simulations. The middle row (**b**, **e**) represents the difference between the average of 2096–2100 and 2016–2020 for the control simulations. The right row (**c**, **f**) represents the difference between the HOS and CTL simulations averaged over 2096–2100. Note the different scaling between **b** and **e**

In CTL-126, an increase in atmospheric CO$_2$ concentration from 400 to 467 ppm in the 2050s is found, after which the concentration decreases to 432 ppm in 2100 (Fig. 1c). This is accompanied by an increase in global mean surface temperature (GMST) of 1 $^{\circ }$C (Fig. 1b), and an AMOC decrease from 17 Sv in 2015 to 9 Sv in 2100 (Fig. 1a). The weakening of the AMOC results in a cooling of the North Atlantic Ocean, while the rest of the Earth warms with largest temperature increases found near the poles (Fig. 2a, b) as a response to the increase in greenhouse gas concentrations. In the water cycle we see a southward shift of the Pacific InterTropical Convergence Zone (ITCZ) of a few degrees (Fig. S4a, b). Furthermore, wind fields in the Northern Hemisphere show a small weakening, whereas in the Southern Hemisphere the winds intensify (Fig. S5a, b).

In CTL-585, the emissions increase the atmospheric CO$_2$ concentration from 400 to 1094 ppm in 2100 (Fig. 1c) which results in a GMST warming of 5 $^{\circ }$C (Fig. 1b). The AMOC weakens from 17 to 7 Sv (Fig. 1a), which leads to a region without warming in the North Atlantic, whereas we see strong warming everywhere else (Fig. 2d, e). There is a strong southward shift of the ITCZ in the Pacific and a moderate shift in the Atlantic Ocean (Fig. S4d, e). The changes in the wind field show similar patterns as CTL-126 but with a larger amplitude (Fig. S5d, e).

The net effect of the AMOC weakening (i.e. HOS minus CTL) is shown in Fig. 1d–f. In the year 2100, atmospheric CO$_2$ concentrations are 2.6 and 4.2 ppm higher in HOS-126 and HOS-585 compared to their respective CTL simulations. In both HOS simulations the AMOC quickly weakens from 17 Sv in 2015 to 6 Sv in 2045 after which the AMOC weakening starts to level off until the AMOC is weaker than 4 Sv in 2100 (Fig. 1d). This strong response to freshwater forcing of the AMOC being independent of greenhouse gas emissions is also found in other studies where a similar freshwater forcing is used (e.g. Jackson et al. 2023). Due to the AMOC weakening we observe a relative cooling of (locally) more than 3 $^{\circ }$C in the Northern Hemisphere and warming in the Southern Hemisphere (Fig. 2c, f) (i.e. the bipolar seesaw). The cooling in the Northern Hemisphere results into an increase in sea-ice cover of the Arctic Ocean (Fig. 5 and S6), which for HOS-126 persists throughout the entire simulation period. The AMOC weakening also results into a stronger southward shift of the ITCZ in both the Pacific and Atlantic Ocean (Fig. S4c, f), and winds are relatively intensified in the Northern Hemisphere and weakened in the Southern Hemisphere (Fig. S5c, f), with a stronger response in SSP5-8.5.

### Marine carbon cycle response

**Fig. 3:**
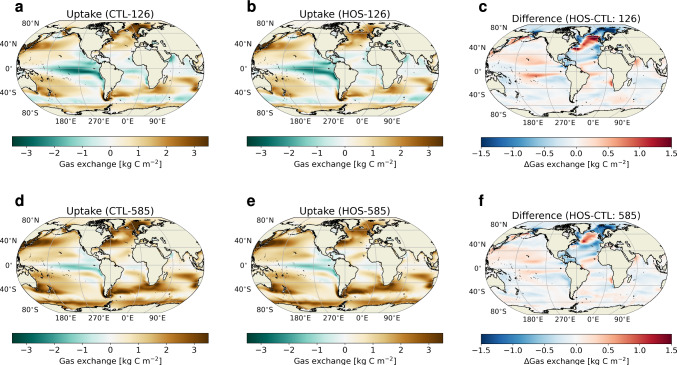
Results for the oceanic CO$_2$ uptake integrated over the entire simulation period in kg C m$^{-2}$. The top row (**a**–**c**) represents SSP1-2.6 and the bottom row (**d**–**f**) represents SSP5-8.5. The left column (**a**, **d**) represents the uptake in the control simulations, the middle column (**b**, **e**) the uptake in the HOS simulations, and the right column (**c**, **f**) the difference between the HOS and CTL simulations. In **a**, **b**, **d**, and **e** positive values (brown colors) represent net uptake, and negative values (blue colors) represent net outgassing

In CTL-126 we see that, integrated over the entire simulation period, there are regions in the ocean with net carbon uptake, and net carbon outgassing (Fig. 3a). The Southern Ocean between 45 and 60$^{\circ }$ S, and the equatorial Pacific Ocean, are regions of carbon release from the ocean to the atmosphere. The region of strongest outgassing in the Pacific is located in the upwelling regions on the eastern side of the basin. Carbon uptake generally occurs in the rest of the ocean with the strongest uptake located in the Sea of Japan and the high latitude North Atlantic Ocean. Looking at the development over time (Fig. S8a, b) we see a negative trend over almost the entire ocean, meaning regions which take up carbon in the beginning of the simulation have lower uptake at the end, and regions which emit carbon in 2015 emit more carbon at the end of the simulation. Some regions, e.g. in the Southern Ocean, shift from a carbon uptake region to a region of outgassing.

In CTL-585, also integrated over the simulation period, only the eastern equatorial Pacific shows strong outgassing (Fig. 3d). In the other equatorial basins, there are also some small patches that show net outgassing, but the rest of the ocean shows net carbon uptake. Except for the high latitude North Atlantic Ocean and some small other regions, we see a positive trend (Fig. S8d, e), meaning that regions that take up carbon in the beginning, take up more carbon at the end of the simulation, and regions which show outgassing in the beginning show either reduced outgassing or go from being a region of outgassing to a region of CO$_2$ uptake. A remarkable region is the high latitude North Atlantic Ocean where the flux from the atmosphere into the ocean strongly decreases while atmospheric pCO$_2$ almost triples.

**Fig. 4:**
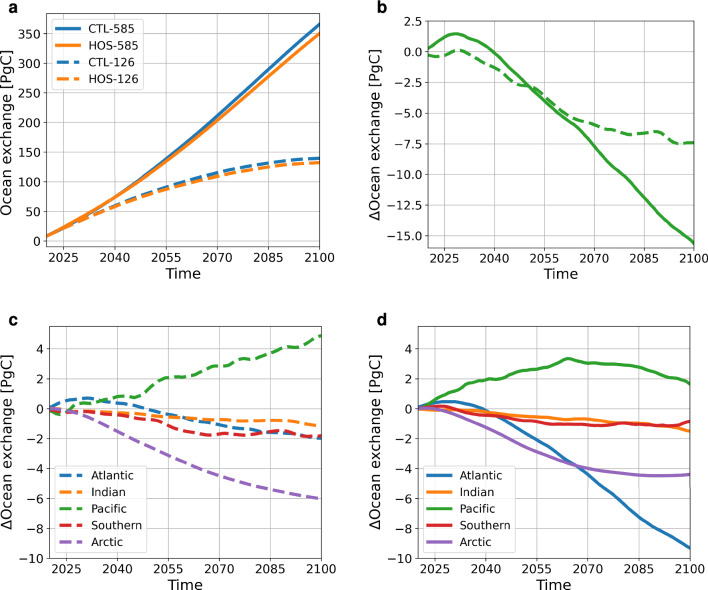
**a** Cumulative uptake of CO$_2$ in the ocean from 2020 onward in PgC. **b** The difference in the cumulative oceanic CO$_2$ uptake between the HOS and CTL simulations. **c** Difference in the cumulative oceanic CO$_2$ uptake between the HOS and CTL simulations in SSP1-2.6 for different ocean basins. **d** As in (**c**) but for SSP5-8.5. In (**a**) blue lines represent the CTL simulations, and the orange lines the HOS simulations. In all subplots dashed lines represent SSP1-2.6 and solid lines SSP5-8.5. Negative values in **b**–**d** represent reduced uptake in the HOS simulations compared to the CTL simulations. Results are smoothed with a 5 year moving mean

Looking at the response to the AMOC weakening, i.e. HOS-CTL, we see that the spatial pattern of regions that have increased or decreased exchange with the atmosphere is very similar for SSP1-2.6 as for SSP5-8.5 (Fig. 3c, f). In total, the ocean takes up 7.4 PgC less due to the AMOC weakening in SSP1-2.6 and 15.6 PgC less in SSP5-8.5 (Fig. 4a, b).

Even though the climate system changes a lot due to the AMOC weakening, the CO$_2$ uptake of the ocean does not change a lot because of compensating effects. To obtain a better understanding of the mechanisms behind the reduced uptake, we have divided the ocean into five basins: the Arctic (north of 66$^{\circ }$ N), the Southern (south of 35$^{\circ }$ S), the Atlantic, Pacific and Indian Ocean (Fig. 4c, d). In the response (i.e. HOS-CTL), the strength of the response differs between the emission scenarios. However, we find that all basins have the same sign, i.e. in both emission scenarios the Pacific takes up more carbon, and the other basins take up less.

**Fig. 5:**
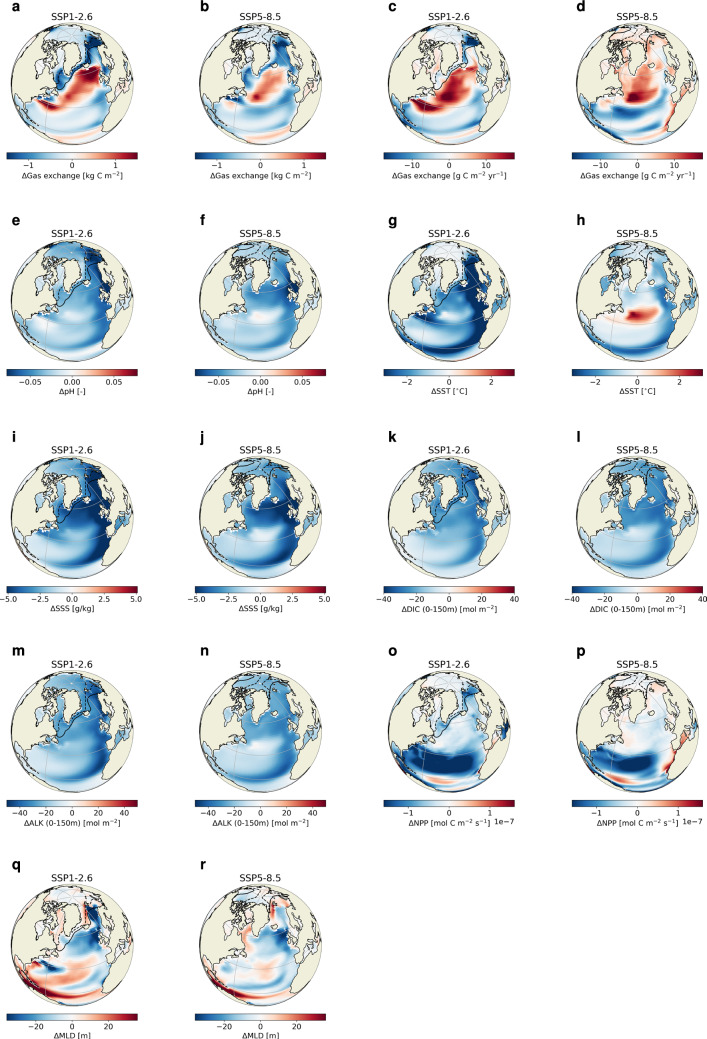
Response of specific variables to the AMOC weakening, i.e. the HOS minus the CTL simulations, zoomed in on the North Atlantic and Arctic Ocean. Columns 1 and 3 represent SSP1-2.6 and columns 2 and 4 SSP5-8.5. The black lines represent the 0.15 sea ice fraction averaged over 2096–2100 for the CTL simulation (dashed lines) and HOS simulation (solid lines). Panels **a** and **b** represent integrated ocean uptake over the entire simulation period, the other panels represent averages over 2096–2100. The variables shown here are the gas exchange (**c**, **d**), surface pH (**e**, **f**), SSTs (**g**, **h**), SSSs (**i**, **j**), DIC in the top 150 m (**k**, **l**), Alk in the top 150 m (**m**, **n**), NPP (**o**, **p**), and maximum mixed layer depth (**q**, **r**)

In both emission scenarios the Arctic Ocean shows a decreased uptake ($-$6.0 PgC in SSP1-2.6 and -4.4 PgC in SSP5-8.5), which can be explained by looking at the sea-ice cover (Fig. 5 and S6). The cooling in the Northern Hemisphere following the AMOC weakening in the HOS simulations, increases the sea-ice cover. The increase in sea-ice cover has two effects on the uptake of CO$_2$: (1) it reduces the ocean area available for exchange with the atmosphere; and (2) it increases light limitation and thereby reduces net primary production (NPP; Fig. 5n, o and S9) and the carbon export to the subsurface ocean. In SSP5-8.5 most of the sea ice still disappears due to the strong warming, but in SSP1-2.6 most of the sea ice persists throughout the simulation period, which explains why the Arctic Ocean in SSP1-2.6 responds stronger compared to SSP5-8.5. We also find this effect in the sea-ice covered regions in the North Atlantic (e.g. the Labrador Sea). Both effects mentioned above contribute to the reduced uptake in the Arctic, which is supported by the linear regression on decadal trends of the ice fraction, NPP and gas exchange showing strong, significant relations with R$^2$-values ranging from 0.79 to 0.97 for both emission scenarios. Especially the relations between NPP and the gas exchange are strong: 0.97 and 0.96 for SSP1-2.6 and SSP5-8.5, respectively.

The Pacific Ocean takes up more carbon in the HOS than in the CTL simulations (+4.9 PgC in SSP1-2.6 and +1.7 PgC in SSP5-8.5). To analyze what is happening in the Pacific, we considered three different regions: (1) the North Pacific (20–66$^{\circ }$ N), the Equatorial Pacific (20$^{\circ }$ N–10$^{\circ }$ S), and the South Pacific (10$^{\circ }$–35$^{\circ }$ S). In the North Pacific, we see an increased uptake of CO$_2$ (Fig. 3e, f), and a relative cooling of the surface ocean (Fig. S10) which increases the solubility of CO$_2$. The linear regression analysis shows a weak relation between SSTs and the gas exchange (R$^2$ is 0.26 and 0.20 for SSP1-2.6 and SSP5-8.5). SSS, surface DIC and surface Alk show no relation to the gas exchange, i.e. all have R$^2$ close to zero. A similar, but opposite, response is seen in the South Pacific. Here the surface ocean becomes relatively warmer decreasing the solubility of CO$_2$. Just as in the North Pacific, the statistical relation is weak (R$^2$ is 0.07 and 0.19 for SSP1-2.6 and SSP5-8.5, respectively), but this could also be related to more spatial variation in the South Pacific Ocean carbon uptake. The equatorial Pacific is characterized by a band with reduced uptake and one with increased uptake. This can be related to the stronger southward shift of the ITCZ in the Pacific in HOS compared to the CTL (Fig. S4). Due to this shift, the dilutive fluxes related to net precipitation shift southward, causing relative increases of SSS, surface DIC and surface Alk in the northern section due to reduced precipitation, and relative decreases due to increased precipitation in the southern section (Fig. S8). The changes in precipitation patterns also affect the stratification, with a weaker stratification in the north and a stronger stratification in the south (Fig. S12). The changes in stratification also affect the entrainment of DIC from DIC rich subsurface layers to the surface ocean, with more entrainment in regions with a weaker stratification. The changes in SSS and surface DIC cause the decreased uptake in the northern section and increased uptake in the southern section. This is supported by the linear regression results which show high R$^2$-values in the northern section for the surface DIC—gas exchange (0.58 and 0.86), SSS—gas exchange (0.56 and 0.72), DIC—stratification (0.95 and 0.96), and stratification—precipitation (0.75 and 0.46) relations, where the values are reported for SSP1-2.6 and SSP5-8.5, respectively.

We find the largest difference in carbon uptake ($-$2.0 PgC in SSP1-2.6 and $-$9.3 PgC in SSP5-8.5) in the Atlantic. In the beginning of the simulation period, the HOS simulations take up more carbon compared to the CTL simulations in both emission scenarios (Fig. 4c, d). This is because initially the response is only present in the subpolar North Atlantic. The strong cooling related to the AMOC weakening and the strong freshening due to the freshwater forcing cause an increase in solubility leading to more uptake of CO$_2$ here. As the AMOC weakening signal spreads to the subtropical gyre, we see that the Atlantic uptake is much reduced in the HOS simulations compared to the CTL simulations.

The regions with sea ice show similar behavior as the Arctic Ocean with decreased uptake related to a larger sea-ice cover in the HOS simulations (Fig. 5). In the ice-free subpolar region, an increase in uptake is observed (Fig. 5a, b). The linear regression analysis shows that the trend in gas exchange is determined by the dilution of Alk by the freshwater forcing (R$^2$-values of 0.66 and 0.84 for SSP1-2.6 and SSP5-8.5 respectively). However, the increased uptake cannot be explained by Alk (Fig. 5m, n), since it decreases in the HOS simulations decreasing the uptake capacity of the ocean. This suggests that we should look towards SSS (Fig. 5i, j), SST (Fig. 5g, h) and surface DIC (Fig. 5k, l) to explain the behavior of the subpolar region. Spatially integrated over the region, pH (Fig. 5e, f) is lower in the HOS simulation which suggests that the increased uptake we find is related to an increase of solubility of CO$_2$. This is associated to the initial strong cooling in this region, and the decreases in SSS (Fig. 5i, j and S11) due to the applied freshwater forcing in this region.

In the subtropical region we generally see a decrease in uptake (Fig. 5a, b). To explain this we consider several variables, i.e. SST (Fig. 5g, h and S10), SSS (Fig. 5i, j and S11), DIC (Fig. 5k, l and S14), Alk (Fig. 5m, n and S15) and NPP (Fig. 5o, p and S9), which all show a relative decrease in this region. The net effect of the changes in these variables is a reduction in pH (Fig. 5e, f and S16) and reduced uptake capacity of the ocean. This is supported by the linear regression analysis between the decadal trends in the gas exchange and surface pH with R$^2$-values of 0.82 and 0.93 for SSP1-2.6 and SSP5-8.5, respectively. The decrease in pH is caused by the decrease in Alk (R$^2$-values of 0.82 and 0.91), which is related to the freshwater forcing in the subpolar region. The freshwater forcing dilutes surface Alk after which it is advected along the eastern side of the basin southwards into the subtropical gyre. Here it reduces the pH and therefore the uptake capacity.

The Indian Ocean has a relatively weak response and is very similar in size for both emission scenarios with a small decrease in uptake ($-$1.2 PgC in SSP1-2.6 and $-$1.5 PgC in SSP5-8.5). In SSP1$-$2.6 this is related to the relatively warmer SSTs in the HOS simulations (Fig. S10), with an R$^2$-value of 0.057. The Southern Ocean also has a small decrease in uptake, with a larger decrease in SSP1-2.6 ($-$1.8 PgC compared to $-$0.9 PgC in SSP5-8.5). This larger decrease can be explained by the fact that the sea-ice cover is larger in SSP1-2.6 compared to SSP5-8.5 (Fig. S7). In SSP5-8.5 we see more uptake around the sea-ice edge just south of 60$^{\circ }$ S while in SSP1-2.6 there is less uptake (Fig. 8d).

### Terrestrial carbon cycle response

**Fig. 6:**
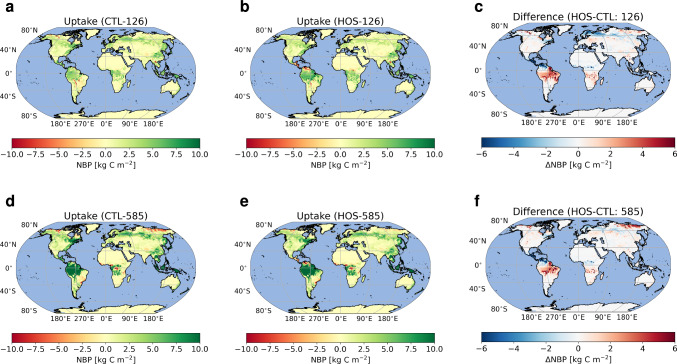
Results for the CO$_2$ exchange with the land integrated over the entire simulation period in kg C m$^{-2}$. The top row (**a**–**c**) represents SSP1-2.6 and the bottom row (**d**–**f**) represents SSP5-8.5. The left column (**a**, **d**) represents the uptake in the control simulations, the middle column (**b**, **e**) the uptake in the HOS simulations, and the right column (**c**, **f**) the difference between the HOS and CTL simulations. In **a**, **b**, **d**, and **e** green colors represent net CO$_2$ uptake by the land, and red colors represent net emissions into the atmosphere

In CTL-126, the terrestrial biosphere, integrated over the entire simulation period, shows a net uptake of CO$_2$ in most regions (Fig. 6a). The Net Biosphere Production (NBP) maxima are located on the equator for the tropical rainforests, the boreal forests in the high latitude Northern Hemisphere, and the eastern United States and China. The few locations that show net emission of CO$_2$ are very local and present in the high latitude Northern Hemisphere, the Tibetan Plateau, South East Asia and South America. If we look at the development over time (Fig. S17a, b) we see that the tropical rainforests have a lower NBP at the end of the simulation. There are some regions that have a higher NBP in 2100, e.g. the boreal forests in Scandinavia.

The response in CTL-585 is very similar to CTL-126 with respect to the spatial pattern, except in central Africa (Fig. 6d). However, the amplitude of the response is much larger due to the CO$_2$ fertilization effect. Especially the tropical rainforests, but also the boreal forests, show more carbon uptake compared to CTL-126. The same is also true for regions that emit carbon, i.e., the region in the high latitude Northern Hemisphere that emits carbon is larger, and the amount of carbon emitted is also higher. The main difference with respect to CTL-126 is a region in the Congo basin which emits CO$_2$ in CTL-585 whereas in CTL-126 it is a region of relatively strong uptake, which is possibly related to increased wildfire activity in this region in SSP5-8.5 (Fig. S18). When we look at the development over time (Fig. S17d, e) we find a completely different pattern in CTL-585 compared with CTL-126. The tropical rainforests show an increase in NBP related to the CO$_2$ fertilization effect whereas northern Siberia shows a decrease related to increased respiration due to permafrost melt (Fig. S20 and S21).

**Fig. 7:**
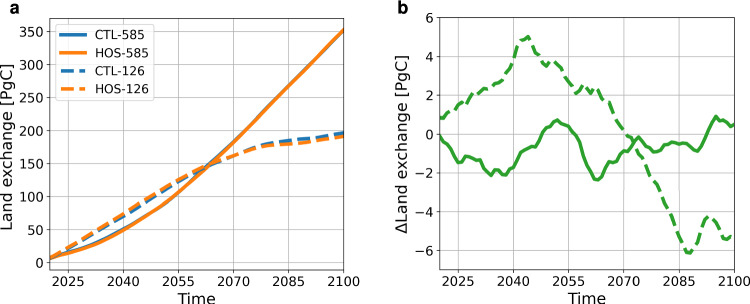
**a** Cumulative uptake of CO$_2$ on the land from 2020 onward in PgC. **b** Difference in the cumulative land uptake of CO$_2$ between the HOS and CTL simulations. Blue lines represent the control simulations, and the orange lines the HOS simulations. In both subplots dashed lines represent SSP1-2.6 and solid lines SSP5-8.5. Negative values in b represent reduced uptake in the HOS simulations compared to the CTL simulations. Results are smoothed with a 5 year moving mean

Integrated globally the terrestrial biosphere takes up 5.3 PgC less in SSP1-2.6 and 0.5 PgC more in SSP5-8.5 (Fig. 7) in the HOS simulations compared to the CTL simulations. Figure 7 shows the opposite trends in carbon uptake on the land for the two emission scenarios: in SSP1-2.6 the land takes up more carbon between 2015 and 2040 in the HOS simulation, whereas in SSP5-8.5 the land takes up less in the HOS simulation for the same period. The increased uptake in HOS-126 compared to CTL-126 can be explained by increased NBP in Brazil. For HOS-126, in the first years there is already an increase in precipitation over Brazil boosting the productivity and therefore also the carbon uptake in this region. The strong decrease in precipitation (related to the ITCZ shift) in Central America only starts in the 2030s and does not influence the response in the first two decades of the simulation period. In HOS-585 the increase in precipitation of Brazil is delayed compared to HOS-126. Additionally, there is a strong carbon outflux in the 2020s in HOS-585 related to fire in the Congo basin (Fig. S18).

Looking at spatial patterns of the cumulative uptake, we see a very similar response to the AMOC weakening (HOS-CTL) for both emission scenarios (Fig. 6c, f). In both emission scenarios we find that the increased southward shift in the ITCZ in the HOS simulations leads to decreased NBP in central America, and increased NBP in Southern America. The linear regression analysis supports this. The decadal trends of NBP and precipitation relate well to each other in both the northern (0.77 and 0.49) and southern section (0.50 and 0.54) for SSP1-2.6 and SSP5-8.5, respectively. A similar shift can be seen in Africa, but with a smaller latitudinal shift and amplitude. The shift and amplitude are slightly stronger in SSP1-2.6. The boreal forests become relatively lower in NBP in the HOS simulations with a larger amplitude in SSP1-2.6. This is because in SSP1-2.6, the forests have lower Gross Primary Production (GPP; Fig. S19) over the course of the century which can be related to the relative cooling in the Northern Hemisphere seen in the HOS simulations (Fig. 2). This relative cooling is stronger in SSP1-2.6, related to the increased sea-ice cover and therefore higher albedo in the Arctic. The R$^2$-values for the linear regression on decadal trends in SAT and GPP over the boreal forests are 0.70 and 0.90 for SSP1-2.6 and SSP5-8.5. Another effect of the Northern Hemispheric cooling is an increase in NBP in the permafrost regions in Siberia and North America in the HOS simulations. The cooling reduces permafrost melt (Fig. S20) and therefore reduces soil respiration (Fig. S21), with a larger amplitude in Siberia for SSP5-8.5. The R$^2$-values for the linear regression on the decadal trends in SATs and soil respiration are 0.79 and 0.61 for SSP1-2.6 and SSP5-8.5, respectively.

### Total response

In total we see an increase of atmospheric CO$_2$ concentration of 2.6 and 4.2 ppm in 2100 in SSP1-2.6 and SSP5-8.5 due to the AMOC weakening (HOS-CTL). In SSP1-2.6 this response is caused due to reduced uptake of the ocean (7.4 PgC) and reduced uptake of the land (5.3 PgC) in 2100. In SSP5-8.5 it is driven by the ocean which has taken up 15.6 PgC less in 2100 in the HOS simulations, whereas the land has taken up 0.5 PgC more in 2100. Eventually the AMOC strength in 2100 has decreased by 5.8 and 3.2 Sv in the HOS simulations compared to the CTL simulations. Under the assumption of linearity, this results in a positive feedback strength of 0.44 ppm Sv$^{-1}$ and 1.3 ppm Sv$^{-1}$ for SSP1-2.6 and SSP5-8.5 respectively. This can be considered a positive feedback since increased CO$_2$ concentrations in future climates are generally associated with a weakening of the AMOC (e.g. Weijer et al. 2020). This AMOC-pCO$_2$ feedback is small on the global scale, due to competing effects but locally large changes in carbon uptake can occur. It is good to note here that the timescale of the feedback can influence the sign of the feedback, since in SSP1-2.6 the CO$_2$ concentration is lower in the HOS simulation compared to the CTL simulation in the first 40 years of the simulation (Fig. 1c,f).

**Fig. 8:**
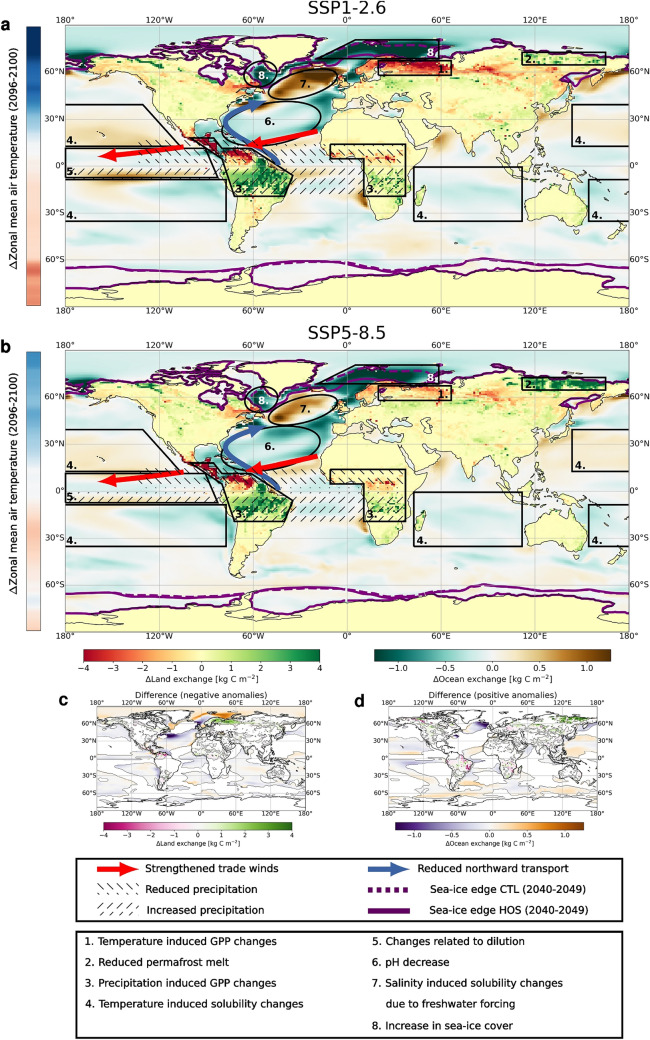
Summarizing figure with dominant mechanisms included for SSP1-2.6 (**a**) and SSP5-8.5 (**b**). **a** and **b** represent results from HOS minus the CTL simulations. The sea-ice edge is taken as where the ice fraction is 0.25 and denoted by the purple lines, where dashed lines represent the CTL simulations and solid lines the HOS simulations. The bar at the left shows the difference in zonal mean surface air temperature averaged over 2096–2100 between HOS and CTL. The scaling of this bar is between $-$2.5 $^{\circ }$C (dark blue) and 2.5 $^{\circ }$C (dark red). **c** The difference between SSP5-8.5 (**b**) and SSP1-2.6 (**a**) for the regions where (**b**) is negative. Negative values represent a higher negative anomaly in SSP5-8.5 compared to SSP1-2.6. (**d**) as in (**c**) but for positive anomalies. Positive values represent a higher positive anomaly in SSP5-8.5 compared to SSP1-2.6. The color bars in (**c**) and (**d**) apply to both subfigures

Figure 8 gives an overview of the most important climate changes and how the marine and terrestrial respond to these changes. In Fig. 8c, d the difference between SSP1$-$2.6 and 5$-$8.5 is highlighted. In the terrestrial biosphere the prime effect of the AMOC weakening is the southward shift of the GPP maxima in the tropical rainforests (Fig. S19). Though this could potentially have beneficial effects for the southern regions, it could have detrimental effects for the northern regions (e.g. the Sahel region) and could for example increase the latitudinal extent of the Sahara desert. This shift, caused by a shift in precipitation (Fig. S4), also has effects for the probability of wildfires (Fig. S18), which can increase in regions with reduced precipitation. We cannot conclude whether the AMOC weakening would result into a collapse of the Amazonian rainforests or an increase in the Sahara desert since the model is used without a dynamic vegetation model. In the ocean, a decrease in NPP (Fig. S9) and surface nutrient concentrations (Fig. S13) occurs. The changes in NPP can have effects on the entire food web and thereby have a negative impact on ecosystems and ecosystem functions. If the trend of the surface ocean becoming more depleted of nutrients continues, this might drive a large decline in NPP for the coming centuries. Another important effect of the AMOC weakening is increased ocean acidification (i.e. a decrease in pH; Fig. S16). Lower pH values increase the stress on calcifying organisms and reduces the uptake capacity of the ocean, which might increase the AMOC-pCO$_2$ feedback strength on longer timescales. However, the strongest ocean acidification observed in the simulations, i.e. the decrease in pH in the North Atlantic and Arctic, is related to the freshwater forcing. Therefore it is difficult to determine how relevant this effect will be in these regions.

In many climate and carbon cycle variables we see a similar response in spatial pattern, but sometimes with a slightly different amplitude (Fig. 8c, d). In the terrestrial biosphere, the main differences are seen in the boreal forests in Scandinavia and Russia (box 1 in Fig. 8), and in the Siberian permafrost regions (box 2). The difference in the boreal forests can be explained by looking at the temperature differences between the HOS and CTL simulations. In SSP1-2.6, the northern hemisphere cools more, which causes increased GPP reduction in the boreal forests. For the permafrost region we find a stronger response in SSP5-8.5, because in SSP1-2.6 there is not much permafrost melt in the CTL simulation; therefore the additional cooling in the HOS simulation does not have a large effect on the permafrost melt. In the ocean, we find the largest changes in the subpolar North Atlantic and the Arctic sea-ice regions (boxes 7 and 8 in Fig. 8). In the subpolar region there is a relatively stronger decrease in SSS and SST (Fig. S10 and S11) in SSP1-2.6 compared to 5–8.5 leading to a larger increase in solubility of CO$_2$ and therefore more uptake. Because of the increased cooling, and lower background temperatures in SSP1-2.6, sea-ice cover does not diminish over the simulation whereas in SSP5-8.5 we see in both simulations a strong reduction in sea-ice cover (Fig. S6). This is the reason why we see a stronger reduction in the Arctic in SSP1-2.6.

## Summary and discussion

In this study, we have investigated the carbon cycle response to a weakening of the Atlantic Meridional Overturning Circulation (AMOC) under climate change scenarios. We did this by forcing a state-of-the-art Earth System Model, the Community Earth System Model v2 (CESM2), on a nominal 1$^{\circ }$ resolution with emissions from two different SSP scenarios (SSP1-2.6 and SSP5-8.5) and an additional freshwater flux in the North Atlantic to artificially decrease the AMOC. To our knowledge, this is the first study that utilizes a model of this high complexity with a horizontal resolution of 1$^{\circ }$ to study the effects of an AMOC weakening on the carbon cycle. We find a positive feedback in both emission scenarios of 0.44 and 1.3 ppm Sv$^{-1}$ for SSP1-2.6 and SSP5-8.5, respectively. The response in SSP1-2.6 is driven by both the land and ocean carbon reservoirs, whereas in SSP5-8.5 it is driven solely by the ocean. The response is small, being the effect of many compensating effects over both the land and the ocean. Looking at regional response patterns, both emission scenarios show similar behavior in many climate and carbon cycle variables. In absolute numbers, the response is stronger in SSP5-8.5, but when the high CO$_2$ concentrations are taken into account, the relative response is actually weaker in SSP5-8.5 compared to SSP1-2.6.

Our simulations show the climate response to an AMOC weakening, such as a southward shift of the ITCZ and the bipolar seesaw, similar to many previous studies (Obata 2007; Zickfeld et al. 2008; Orihuela-Pinto et al. 2022). The AMOC weakening in our simulations follows a very similar trajectory as in Orihuela-Pinto et al. (2022), which used an older version of CESM (i.e. v1.2) under pre-industrial boundary conditions. In our study, the AMOC weakening results in a small increase in atmospheric CO$_2$ concentrations. This small effect, especially on the multi-decadal to centennial timescales assessed here, was also found in more idealized models (e.g. Zickfeld et al. 2008; Nielsen et al. 2019; Gottschalk et al. 2019), but as described in Gottschalk et al. (2019) the relative response of the ocean and land reservoirs are dependent on climatic boundary conditions and the used model. It also agrees well with Winton et al. (2013) where they assess the effect of changing ocean circulation on heat and carbon uptake and also find a small decrease in carbon uptake as a response to changing ocean currents. Here, we have used a member of the newest generation of Earth System Models with a relatively high spatial resolution (i.e. nominal 1$^{\circ } \times$ 1$^{\circ }$ ocean grid). When considering studies with induced AMOC weakening we find, integrated over the entire ocean, a similar response as in Zickfeld et al. (2008), and spatially as in Obata (2007), though local differences remain which can be attributed to the use of a higher resolution, and a more complex model in our study. It is also possible to collapse the AMOC without an additional freshwater forcing. In Nielsen et al. (2019) they used such an alternative method under Pleistocene conditions, which resulted in a much slower response in the ocean compared to our simulations. The response of the terrestrial biosphere, especially the changes related to the southward shift of the ITCZ, is also similar to that of previous studies using static vegetation (e.g. Obata 2007; Nielsen et al. 2019). In Köhler et al. (2005) a dynamic vegetation model is used, and they show that an AMOC collapse affects vegetation type. This leads to reduced carbon storage in the high latitudes and increased carbon storage in the Northern Hemisphere midlatitudes. This dynamic behavior is not captured in our simulations and unfortunately, it is not possible to assess what the effect of dynamic vegetation would be based on Köhler et al. (2005) since they consider Pleistocene conditions.

The result that the pattern of the carbon cycle response to an AMOC weakening is independent of the cumulative CO$_2$ emissions on multi-decadal to centennial timescales has been shown before. In Zickfeld et al. (2008), for example, the marine carbon cycle remains independent on the used emission scenario for the first 200 years of their simulation, and for the terrestrial carbon cycle this is 150 years. After this period the different emissions start to diverge, though the qualitative behavior remains similar. In our simulations, globally integrated variables show little change as a response to the AMOC weakening. However, on regional scales the effects of an AMOC weakening can be large, e.g. SATs can decrease or increase by more than 3 $^{\circ }$C locally (Fig. 2) and some regions become much drier and other see a large increase in precipitation (Fig. S4). These changing climate conditions, on top of already greenhouse gas driven climate change, require climate adaptation which might be difficult to achieve in such a short time frame (i.e. decades). The climate changes associated to an AMOC weakening also cause changes in the carbon cycle. Such changes can increase, for example, desertification and reduce (but also increase) crop yields. This may lead locally to increased food stress, potentially leading to more frequent and more severe famines. The changes in the ocean can lead to more frequent marine heatwaves in the Southern Hemisphere due to the warming, and reduced (global) NPP due to changing nutrient distributions, which might impact food web dynamics and ecosystem function. However, due to the cooling effect of the bipolar seesaw we would can also expect a (relative) reduction in marine heatwaves in the Northern Hemisphere. These effects show that an AMOC collapse can have local effects that have a beneficiary impact or a detrimental impact on the terrestrial and marine biospheres.

Interestingly, the relative effects on multi-decadal timescales are independent to the (cumulative) greenhouse gas emissions. This means that the uncertainty around the effects of a possible AMOC collapse or weakening is not related to past emissions. However, in a future climate without AMOC weakening, emissions do have an influence on when the AMOC might collapse. Furthermore, the small positive feedback found in this study might make the AMOC more likely to tip earlier. Even though on these timescales the relative effects are not dependent on the greenhouse gas emissions, this might be different on intermediate (multi-centennial to millennial) timescales. Since ocean circulation changes, and especially the meridional overturning circulation, is especially dominant in the Earth System on the intermediate timescales, we can expect the most important effects to occur in this time frame. We find, for example, that the surface ocean is becoming more depleted of nutrients (Fig. S13), which might depress NPP for centuries.

Other long term effects that might be relevant are tipping cascades (e.g. Dekker et al. 2018), meaning that a collapse of the AMOC could set off an other tipping element in the Earth System. In our simulations, we find decreasing temperatures in the Northern Hemisphere due to the AMOC weakening, which reduces the probability of tipping for example melting of the Greenland Ice Sheet, Arctic sea ice, and Northern Hemispheric permafrost. However, due to the bipolar seesaw, the Southern Hemisphere becomes warmer, which might increase the probability of tipping the Antarctic Ice Sheets. Another tipping point connected to the AMOC is the die off of the Amazonian rainforest. Because we do not use a dynamic vegetation model in this study, we cannot investigate whether the AMOC weakening in our simulations would lead to such a die off.

By using a low and a high emission scenario we have tried to cover uncertainties regarding future emissions. However, we have only used one Earth System Model, which means that the results presented here could be model dependent. Especially ocean productivity shows large spread in the CMIP6 ensemble, which can influence the uptake capacity of the ocean. Another bias in Earth System Models is a too stable AMOC, meaning we need a large freshwater flux in the North Atlantic Ocean to weaken the AMOC. This flux is generally too high to represent for example Greenland Ice Sheet melt, but necessary to achieve a weakened AMOC. This large freshwater flux also leads to freshening of the surface ocean in the subpolar gyre, and dilutes both surface DIC and surface Alk which influences the carbonate chemistry and carbon uptake capacity unrealistically. These effects are present in both the subpolar and subtropical gyres in the North Atlantic Ocean. We have not taken this effect into account explicitly, but it could potentially result in a different uptake capacity of the ocean, and therefore a different feedback strength.

Finally, we have shown in a relatively high resolution, state-of-the-art Earth System Model, that the spatial pattern of the carbon cycle response to an AMOC weakening is not dependent on cumulative CO$_2$ emissions. As a follow up study it would be interesting to see what happens on multi-centennial and longer timescales, and what the pCO$_2$ response would be under an AMOC recovery. Though not analyzed thoroughly, NPP in the ocean shows large decreases due to the AMOC weakening. This could affect food web dynamics in the ocean with possible (detrimental) changes in fishery yields, food securities and income. These ecosystem and socio-economic effects are worth investigating, to see how a change in the climate system cascades through ecosystems to socio-economic systems.

## Supplementary Information

Below is the link to the electronic supplementary material.

**Supplementary information** This article has Supplementary Information containing additional figures. (pdf 38,038KB)

## Data Availability

Yearly output for the most important variables, data necessary to replicate the figures, and the scripts used for creating the figures can be downloaded from https://doi.org/10.5281/zenodo.8376701 [47].
