# Supplementary material for: Response of atmospheric pCO2 to a strong AMOC weakening under low and high emission scenarios
Source: Clim Dyn. 2024 Jun 6;62(8):7559–74. doi: 10.1007/s00382-024-07295-y (PMC11554733; doi:10.1007/s00382-024-07295-y)
Supplement: Supplementary file 1 — Supplementary information This article has Supplementary Information containing additional figures. (pdf 38,038KB) [file 382_2024_7295_MOESM1_ESM.pdf]

SI for "Response of atmospheric pCO_2 to a strong AMOC weakening under low and high emission scenarios"

Amber A. Boot[1*], Anna S. von der Heydt[1,2] and Henk A. Dijkstra[1,2]

[1*]Institute for Marine and Atmospheric research Utrecht, Department of Physics, Utrecht University, Princetonplein 5, Utrecht, 3584CC, Utrecht, the Netherlands.
[2]Centre for Complex Systems Studies, Utrecht University, Leuvenlaan 4, Utrecht, 3584CE, Utrecht, the Netherlands.

*Corresponding author(s). E-mail(s): a.a.boot@uu.nl;

1 Introduction

This supplementary material includes additional figures of the results.

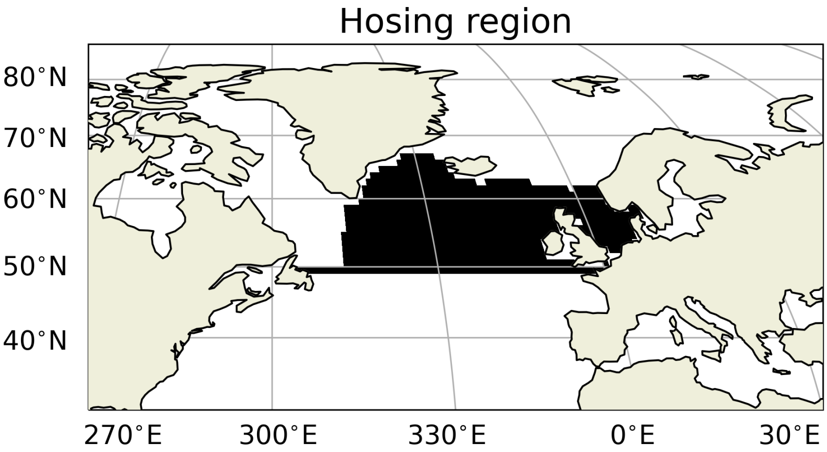

Fig. S1 Region in black corresponds to the region where the freshwater forcing is applied. The freshwater forcing integrated over this region is 0.5 Sv throughout the entire simulation period.

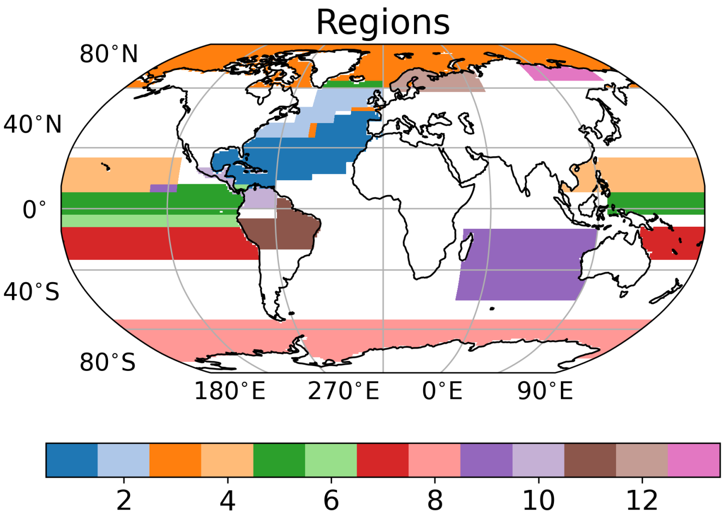

Fig. S2 Regions used for the linear regression mentioned in the main text for the following regions: (1) Subtropical North Atlantic, (2) Subpolar North Atlantic, (3) Arctic, (4) North Pacific, (5) North Equatorial Pacific, (6) South Equatorial Pacific, (7) South Pacific, (8) Southern Ocean, (9) Indian Ocean, (10) northern rainforests, (11) southern rainforests, (12) boreal forests, and (13) permafrost regions. Some grid cells are used in multiple regions, see e.g. in the subpolar North Atlantic where a couple of grid cells are used for both the Arctic region and the subpolar region.

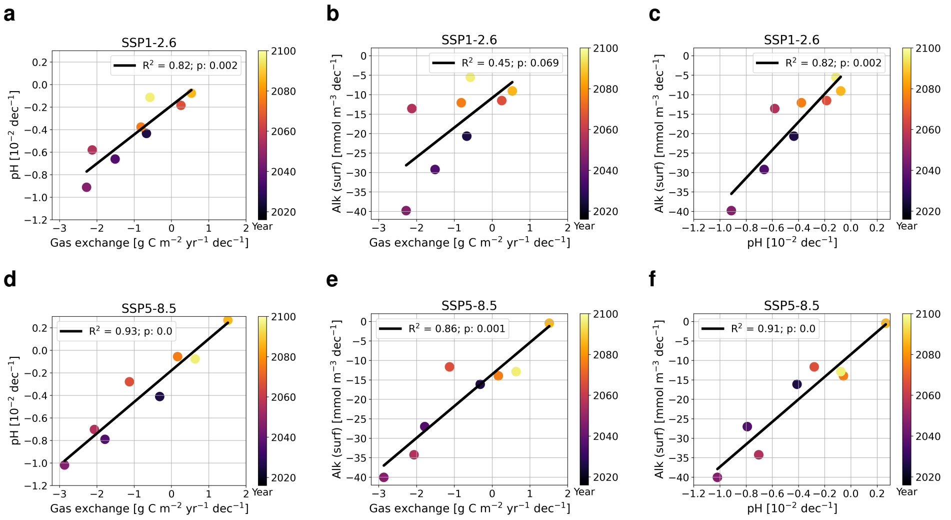

Fig. S3 Linear regression on decadal trends for the Subtropical North Atlantic for the difference between the HOS and CTL simulations for SSP1-2.6 (top row), and SSP5-8.5 (bottom row). (a) Linear regression between the trends in gas exchange in g C m^{-2} dec^{-1} and surface pH in 10^2 dec^{-1}. (b) Linear regression between the trends in gas exchange in g C m^{-2} dec^{-1} and surface alkalinity mmol m^{-3} dec^{-1}. (c) Linear regression between the trends in surface pH in 10^2 dec^{-1} and surface alkalinity mmol m^{-3} dec^{-1}.

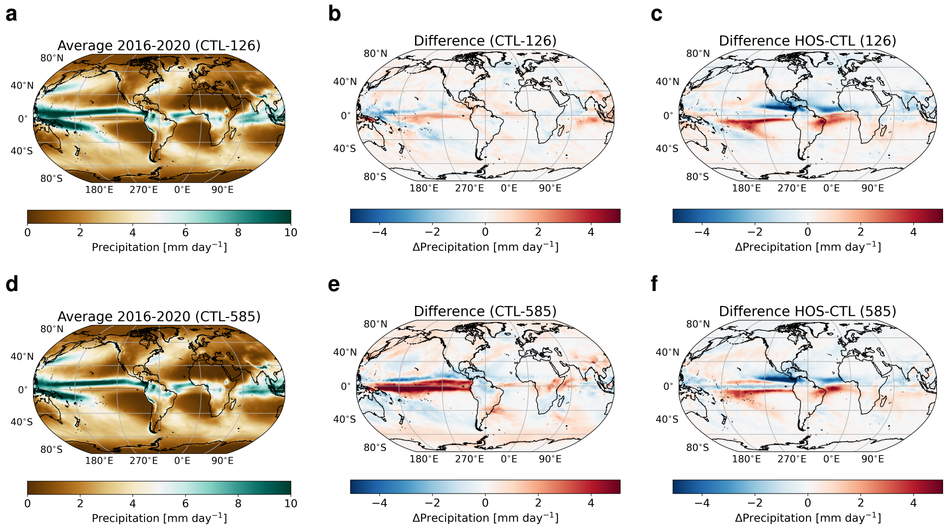

Fig. S4 Results for precipitation in mm day^{-1}. he top row (a-c) is for SSP1-2.6, and the bottom row (d-f) for SSP5-8.5. The left column (a, d) represents the average over 2016-2020 in the control simulations. The middle row (b, e) represents the difference between the average of 2096-2100 and 2016-2020 for the control simulations. The right row (c, f) represents the difference between the hosing and control simulations averaged over 2096-2100. Note the different scaling between b and e.

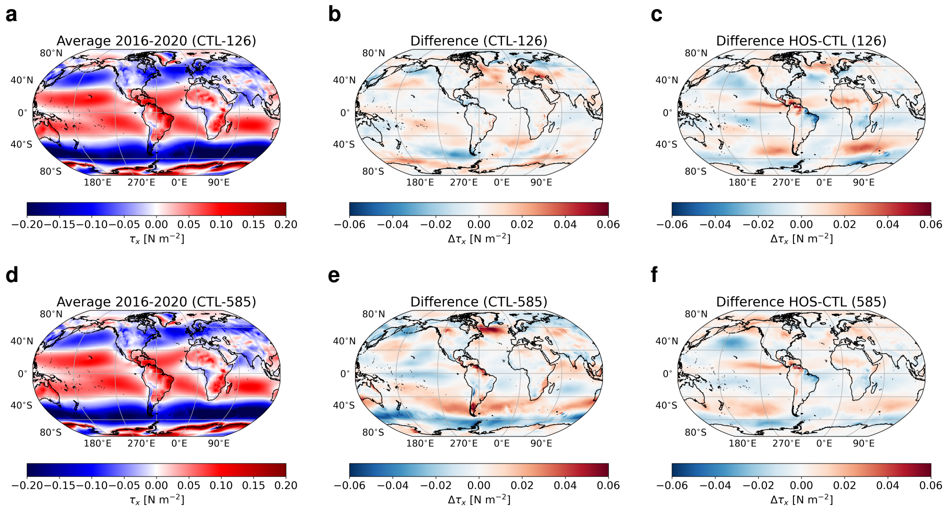

Fig. S5 Results for the zonal wind stress in N m^{-2}. Panels represent the same as in Fig. S4.

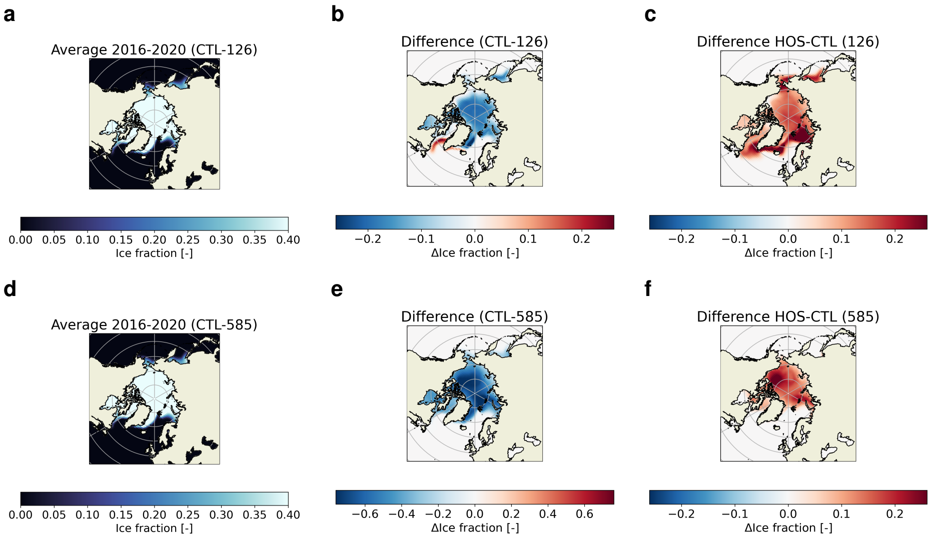

Fig. S6 Results for the ice fraction in the Arctic. Panels represent the same as in Fig. S4. Note the different scaling for e.

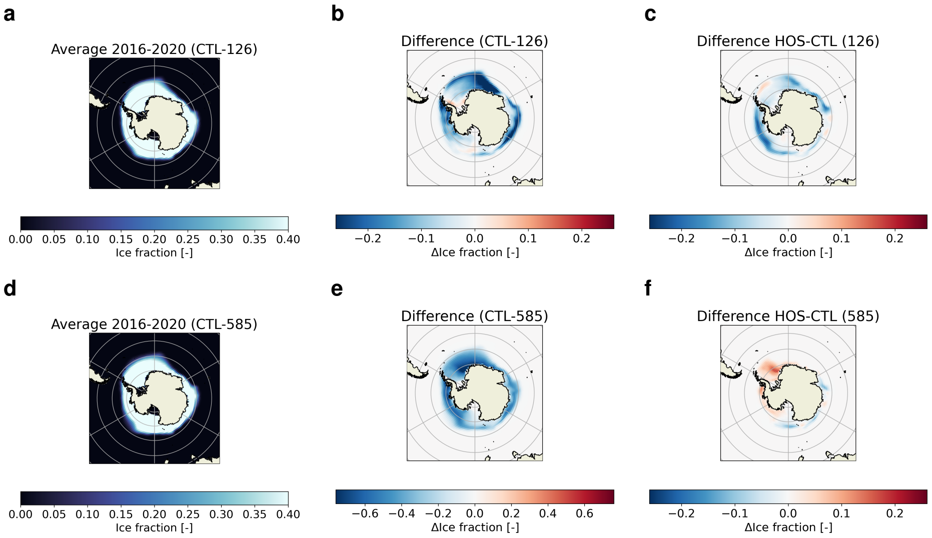

Fig. S7 Results for the ice fraction in the Antarctic. Panels represent the same as in Fig. S4. Note the different scaling for e.

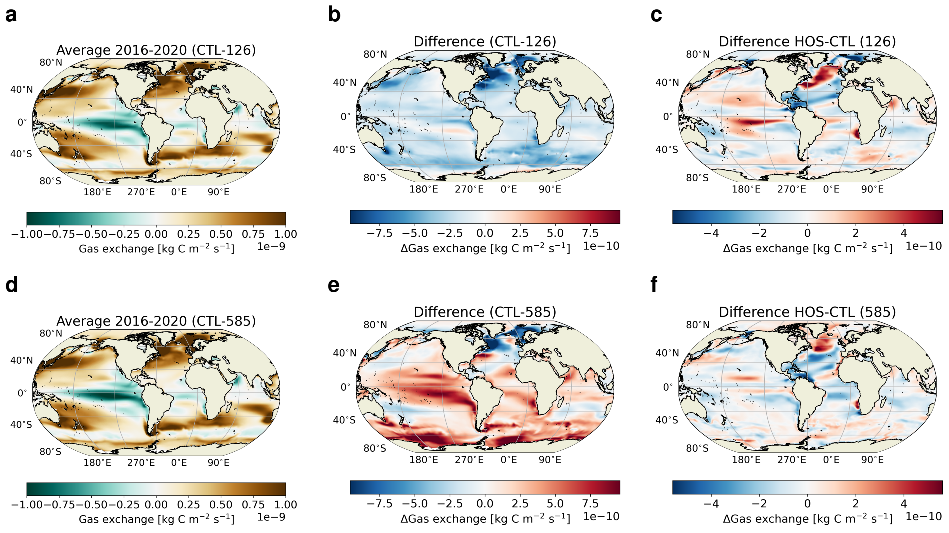

Fig. S8 Results for oceanic CO_2 uptake in kg C m^{-2} s^{-1}. Panels represent the same as in Fig. S4. Positive values (brown colors) in a and d represent uptake by the ocean and negative values (blue colors) represent outgassing.

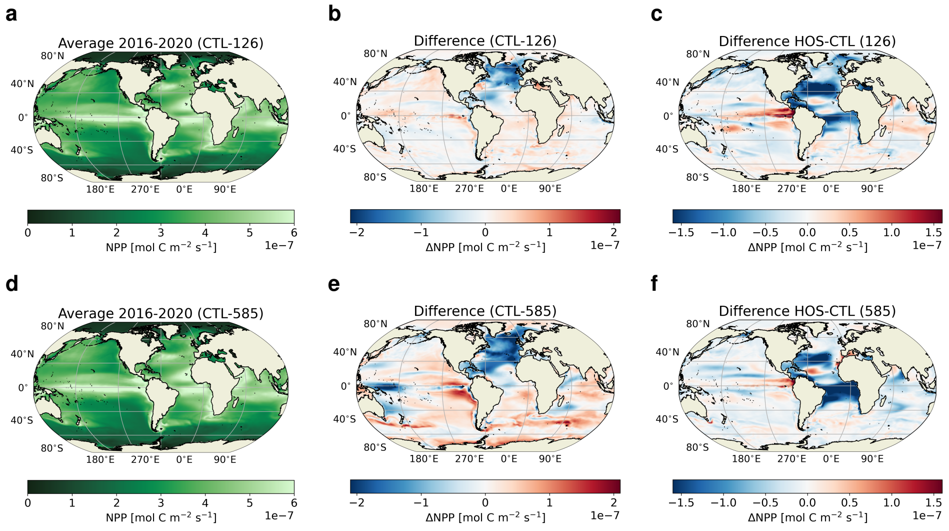

Fig. S9 Results for Net Primary Production (NPP) integrated over the surface layer (0-150 m) in mol m^{-2} s^{-1}. Panels represent the same as in Fig. S4

.

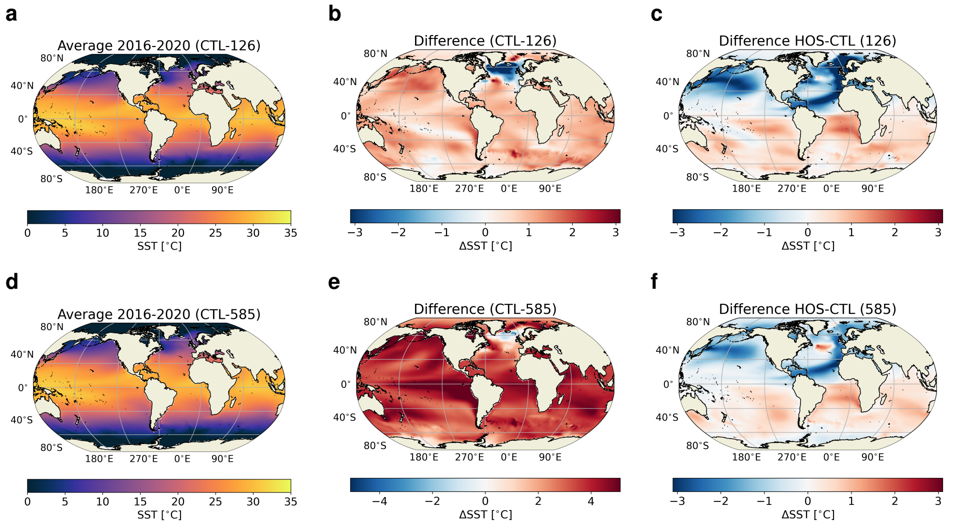

Fig. S10 Results for Sea Surface Temperature (SST) in $°C$. Panels represent the same as in Fig. S4. Note the different scaling in e.

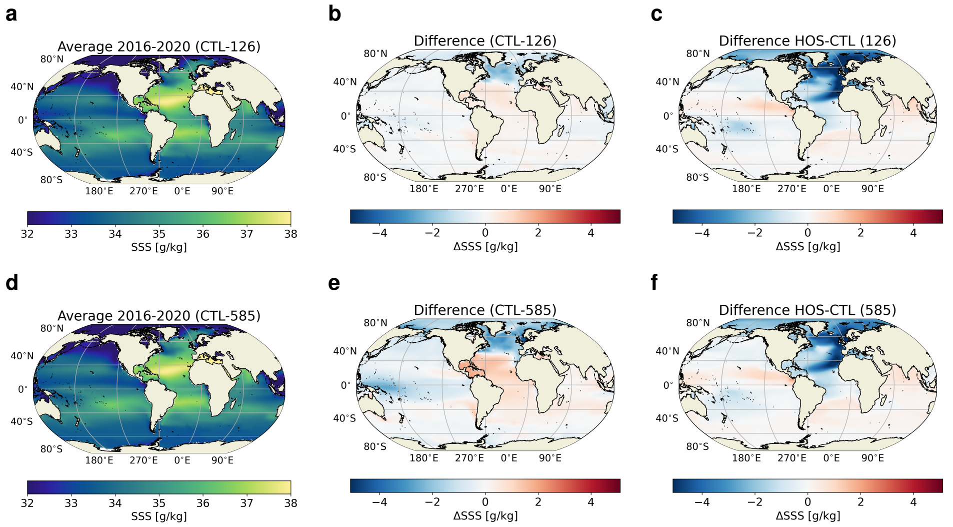

Fig. S11 Results for Sea Surface Salinity (SSS) in g/kg. Panels represent the same as in Fig. S4.

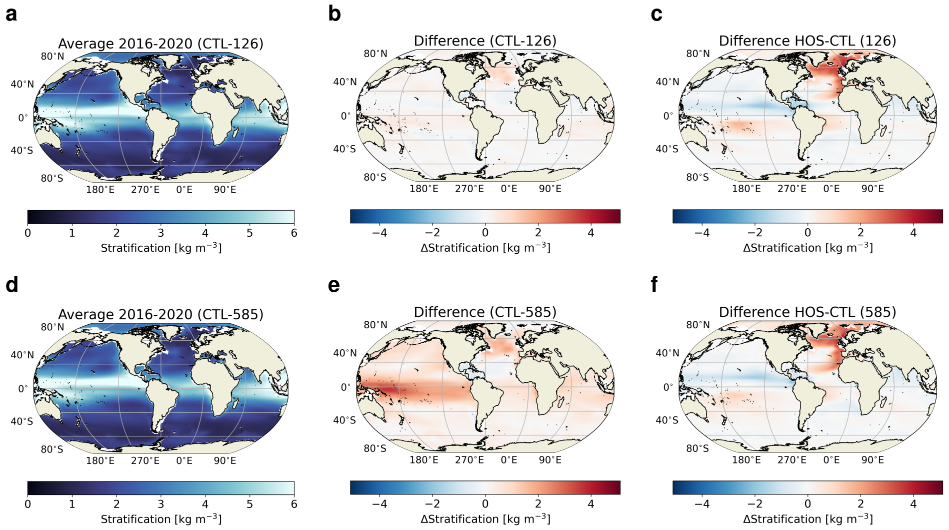

Fig. S12 Results for stratification in kg m^{-3}, where stratification is defined as the density difference between 200 m depth and the surface [?]. Panels represent the same as in Fig. S4.

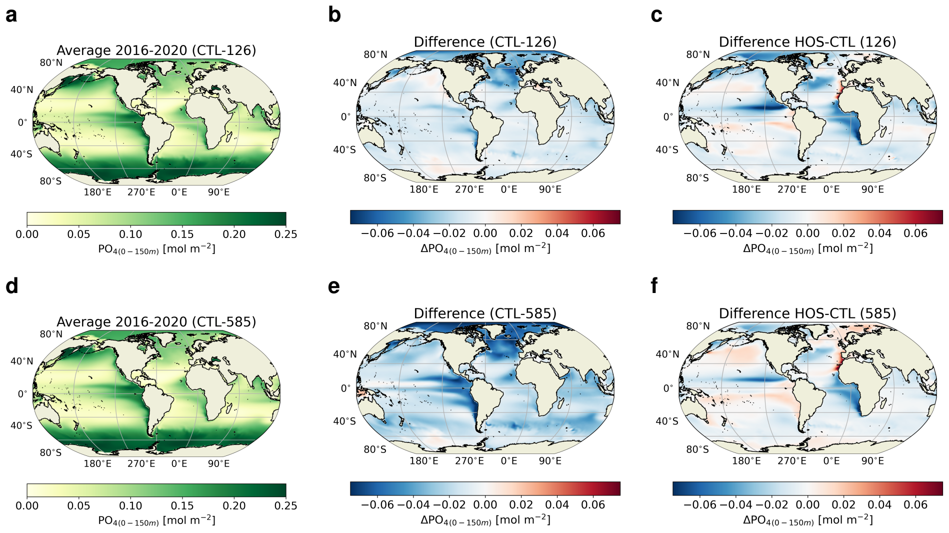

Fig. S13 Results for PO_4 concentrations integrated over the surface layer (0-150 m) in mol m^{-2}. Panels represent the same as in Fig. S4.

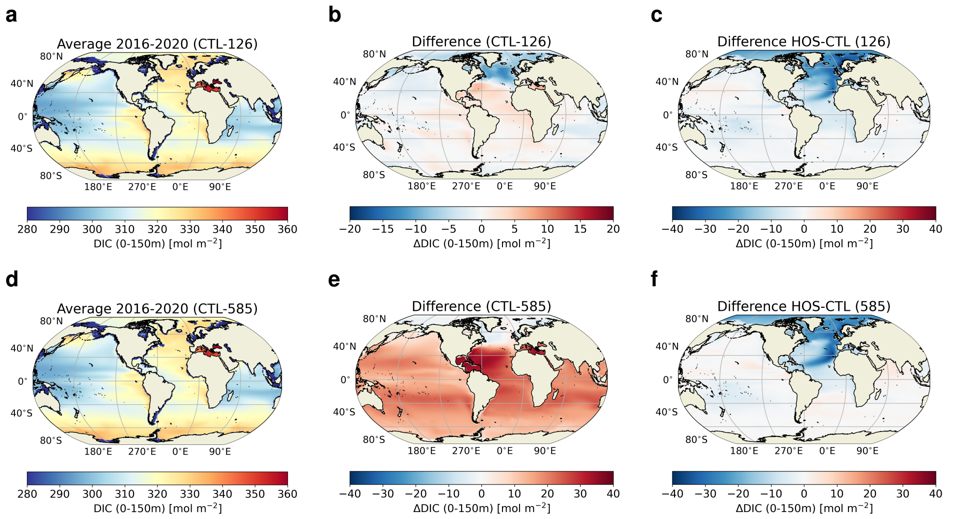

Fig. S14 Results for DIC concentrations integrated over the surface layer (0-150 m) in mol m^{-2}. Panels represent the same as in Fig. S4. Note the different scaling in e.

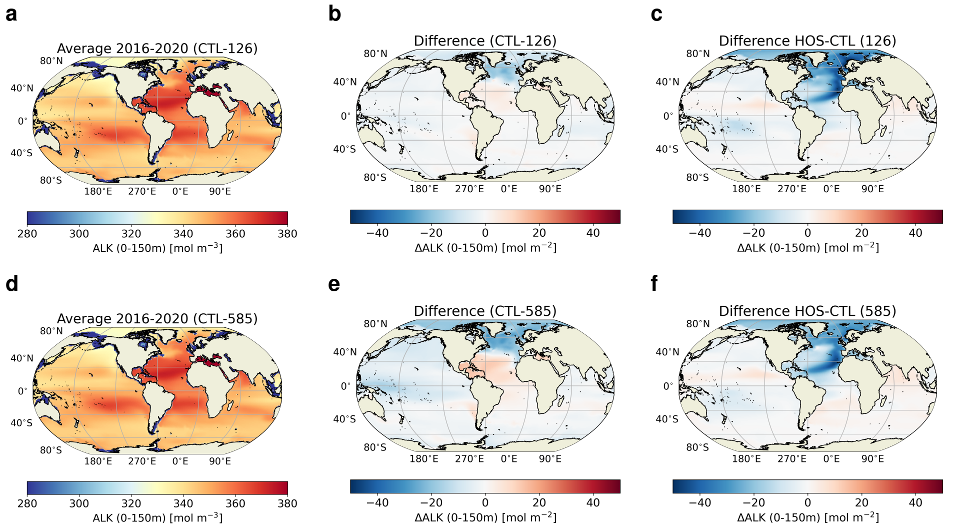

Fig. S15 Results for alkalinity concentrations integrated over the surface layer (0-150 m) in mol m^{-2}. Panels represent the same as in Fig. S4. Note the different scaling in e.

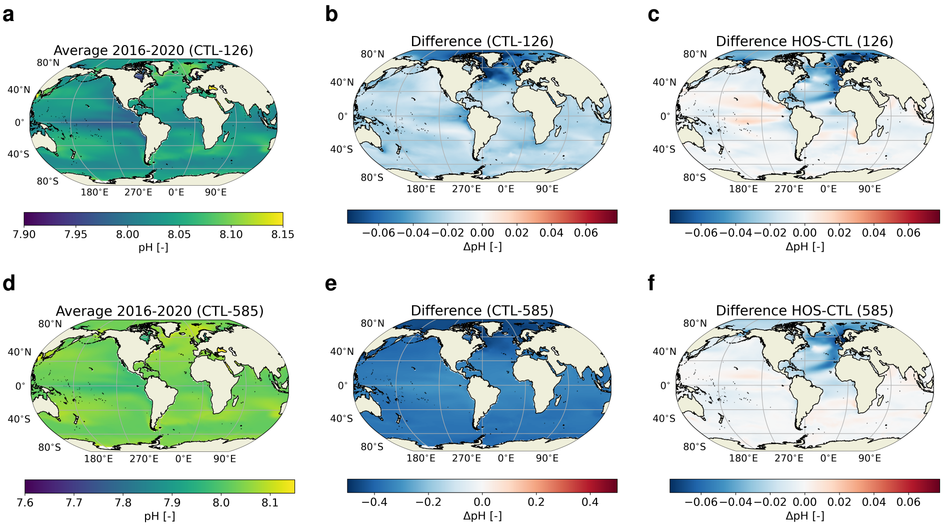

Fig. S16 Results for surface pH. Panels represent the same as in Fig. S4. Note that the scaling of the colorbar is different for the subplots.

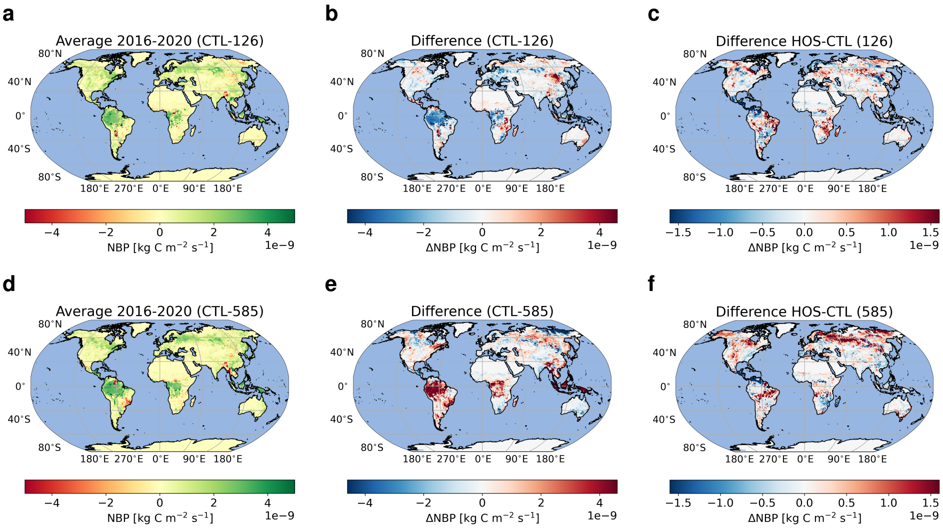

Fig. S17 Results for Net Biosphere Production (NBP) in kg C m^{-2} s^{-1}. Panels represent the same as in Fig. S4. Green colors represent uptake of CO_2 into the land and red colors represent emission of CO_2 to the atmosphere.

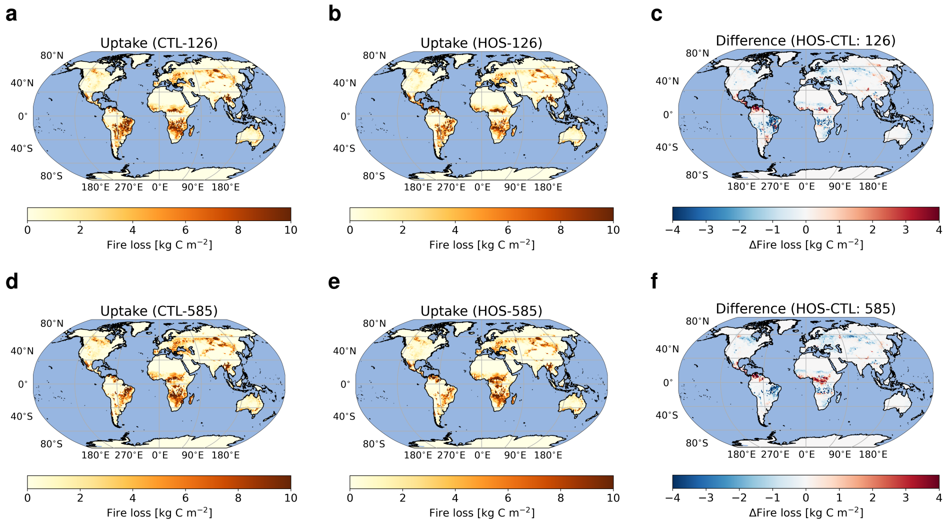

Fig. S18 Results for biomass loss due to fire integrated over the entire simulation period in kg C m^{-2}. The top row (a-c) represents SSP1-2.6 and the bottom row (d-f) represents SSP5-8.5. The left column (a, d) represents the uptake in the control simulations, the middle column (b, e) the uptake in the hosing simulations, and the right column (c, f) the difference between the hosing and control simulations.

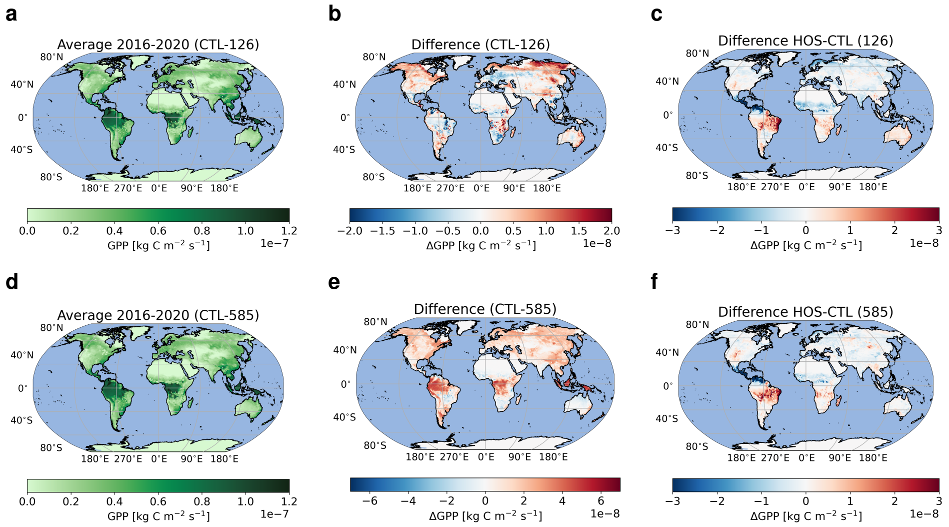

Fig. S19 Results for Gross Primary Production (GPP) in kg C m^{-2} s^{-1}. Panels represent the same as in Fig. S4. Note the different scaling in e.

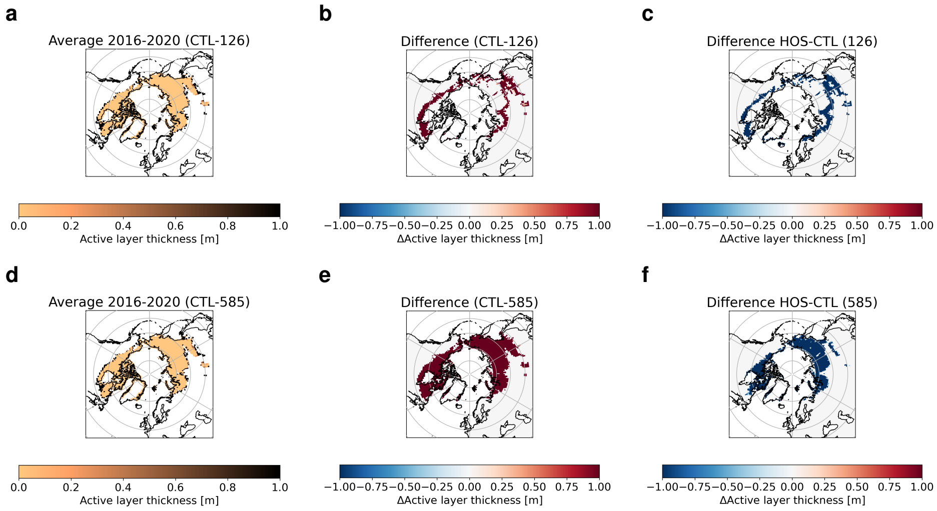

Fig. S20 Results for Active Layer Thickness (ALT) in m, which serves as a proxy for annually minima of (horizontal) permafrost extent. Panels represent the same as in Fig. S4.

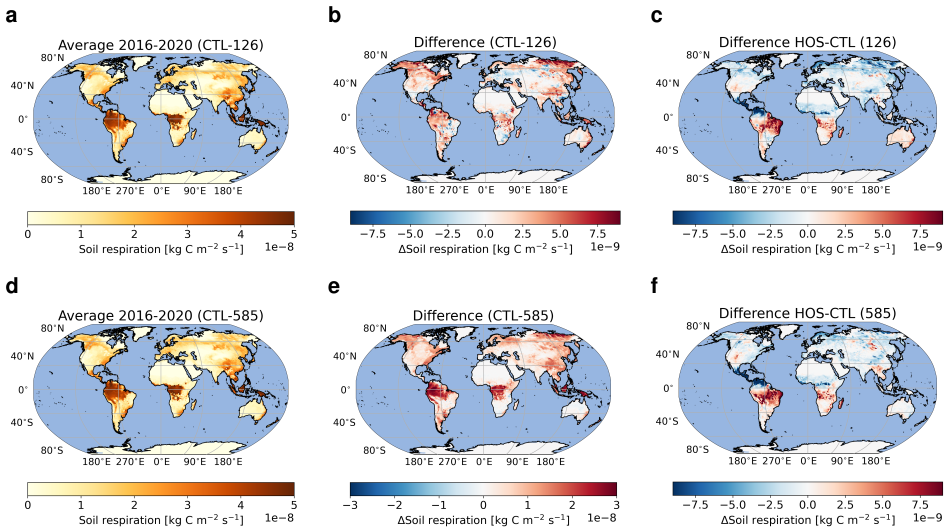

Fig. S21 Results for soil respiration in kg C m^{-2} s^{-1}. Panels represent the same as in Fig. S4. Note the different scaling in e.

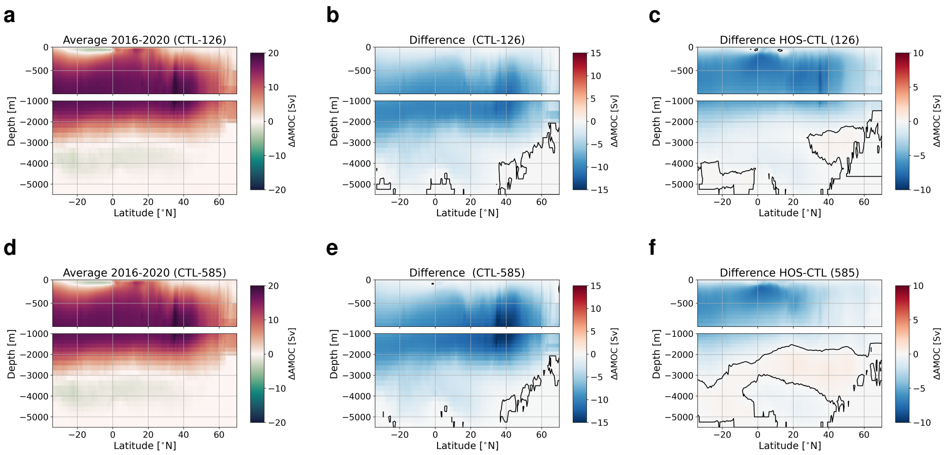

Fig. S22 Results for the Atlantic Meridional Overturning Circulation in Sv. Panels represent the same as in Fig. S4. Black contour lines in b, c, e and f represent the 0 Sv contour. Note the different scaling of the surface ocean (top 1000 m) compared to the deep ocean.
